# Supplementary material for: A Shortcut from Genome to Drug: The Employment of Bioinformatic Tools to Find New Targets for Gastric Cancer Treatment
Source: Pharmaceutics. 2023 Sep 12;15(9):2303. doi: 10.3390/pharmaceutics15092303 (PMC10535099; doi:10.3390/pharmaceutics15092303)

Supplementary Table S1 - List of studies for the construction of the meta-data

Access ID	Samples	Platform
GSE13911	69	Affymetrix Human Genome U133 Plus 2.0 array
GSE54129	132	Affymetrix Human Genome U133 Plus 2.0 array
GSE66229	400	Affymetrix Human Genome U133 Plus 2.0 array

Supplementary Table S2 - List of RNAm, NCBI Reference Sequences and Primer sequences (forward and reverse complement) for the PCR

GENE	CÓDIGO NCBI	SEQUENCE
AJUBA	NM_001289097.2<br>NM_032876.6	F-GATGCTGTGGGATTCCTGGC<br>R-GAACTTCTCCAGCAGGCGAC
CD80	NM_005191.4	F-GGATTGTCATCAGCCCTGCC<br>R-GAGAAAGACCAGCCAGCACC
NOLC1	NM_001284388.2<br>NM_004741.5<br>NM_001284389.2	F-GCTCTCCAAGACCACAAGCC<br>R-CCTCCCTGACCCTTCGGAAT
KNL1	NM_144508.5<br>NM_170589.5	F-CTACCACCCCTTCCAGAGCA<br>R-TGGCGGTAGAGAGGTGGTTT

Supplementary Table S3 – List of top 10 ligands identified by screening MCULE's purchasable library using AutoDock Vina for the AJUBA target, organized in descending order of minimum energy value. Results obtained for prediction of toxicity class, toxicity endpoint models, docking score by DockThor server and final punctuation

MCULE ID	*Docking Score Mcule (Kcal mol⁻¹)*	*Predicted Toxicity Class*	*Toxicity Endpoint Models*	*Docking Score DockThor (Kcal mol⁻¹)*	*Punctuation*
**MCULE-2386589557-0-6***	-7.3	5	Inactive	-8.483	2
**MCULE-8494477087-0-5***	-7.1	5	Inactive	-7.767	1.75
MCULE-8618831675-0-4	-7.1	3	Active		
MCULE-4680685315-0-2	-7.1	4	Active		
MCULE-3694094262-0-3	-7.1	4	Active		
MCULE-8663038790-0-3	-6.9	4	Active		
MCULE-6320162783-0-1	-6.9	4	Active		
MCULE-8680129923-0-1	-6.9	4	Active		
**MCULE-5479669360-0-2***	-6.9	4	Inactive	-7.488	1.50
MCULE-4497233309-0-6	-6.8	3	Active		

*: Compounds selected for the molecular docking step on the DockThor server. Compounds with favorable toxicological properties were chosen as a selection criterion, without prediction for the toxicity endpoint models and with a prediction of toxicity class equal to or greater than 4.

Supplementary Table S4 – List of top 10 ligands identified by screening MCULE's purchasable library using AutoDock Vina for the CD80 target, organized in descending order of minimum energy value. Results obtained for prediction of toxicity class, toxicity endpoint models, docking score by DockThor server and final punctuation

MCULE ID	*Docking Score Mcule (Kcal mol^{-1})*	*Predicted Toxicity Class*	*Toxicity Endpoint Models*	*Docking Score DockThor (Kcal mol^{-1})*	*Punctuation*
MCULE-9029233932-0-3	-6.5	4	Active		
MCULE-2278849636-0-73	-5.8	4	Active		
MCULE-7527853534-0-1	-5.7	3	Active		
MCULE-7110142890-0-1	-5.7	4	Active		
MCULE-4428265531-0-1	-5.7	4	Active		
**MCULE-2625873551-0-8***	-5.6	4	Inactive	-7.387	1.875
**MCULE-9178344200-0-1***	-5.6	4	Inactive	-7.773	2
MCULE-5779193146-0-2	-5.6	3	Active		
**MCULE-4809120560-0-2***	-5.6	4	Inactive	-7.375	1.75
MCULE-2768743746-0-1	-5.6	4	Active		

*: Compounds selected for the molecular docking step on the DockThor server. Compounds with favorable toxicological properties were chosen as a selection criterion, without prediction for the toxicity endpoint models and with a prediction of toxicity class equal to or greater than 4.

Supplementary Table S5– List of top 10 ligands identified by screening MCULE's purchasable library using AutoDock Vina for the NOLC1 target, organized in descending order of minimum energy value. Results obtained for prediction of toxicity class, toxicity endpoint models, docking score by DockThor server and final punctuation

MCULE ID	*Docking Score Mcule (Kcal mol^{-1})*	*Predicted Toxicity Class*	*Toxicity Endpoint Models*	*Docking Score DockThor (Kcal mol^{-1})*	*Punctuation*
MCULE-5664033778-0-1	-8.6	4	Active		
MCULE-1565012771-0-2	-8.0	3	Inactive		
MCULE-3493609194-0-3	-7.8	4	Active		
MCULE-7733662288-0-1	-7.7	4	Active		
MCULE-5092204522-0-3	-7.7	5	Active		
MCULE-7508528499-0-3	-7.6	4	Active		
**MCULE-2888284016-0-4***	-7.6	4	Inactive	-8.502	1.75
**MCULE-5881513100-0-29***	-7.5	4	Inactive	-7.260	1.5
MCULE-6651045318-0-2	-7.5	4	Active		
MCULE-5189774934-0-5	-7.4	4	Active		

*: Compounds selected for the molecular docking step on the DockThor server. Compounds with favorable toxicological properties were chosen as a selection criterion, without prediction for the toxicity endpoint models and with a prediction of toxicity class equal to or greater than 4. Compound MCULE-5881513100-0-29 was selected over MCULE-2888284016-0-4 because the synthesis of this compound has been discontinued by the company Mcule.

Supplementary Figure S1. Enrichment plots of genes were analyzed using Gene Set Enrichment Analysis (GSEA).

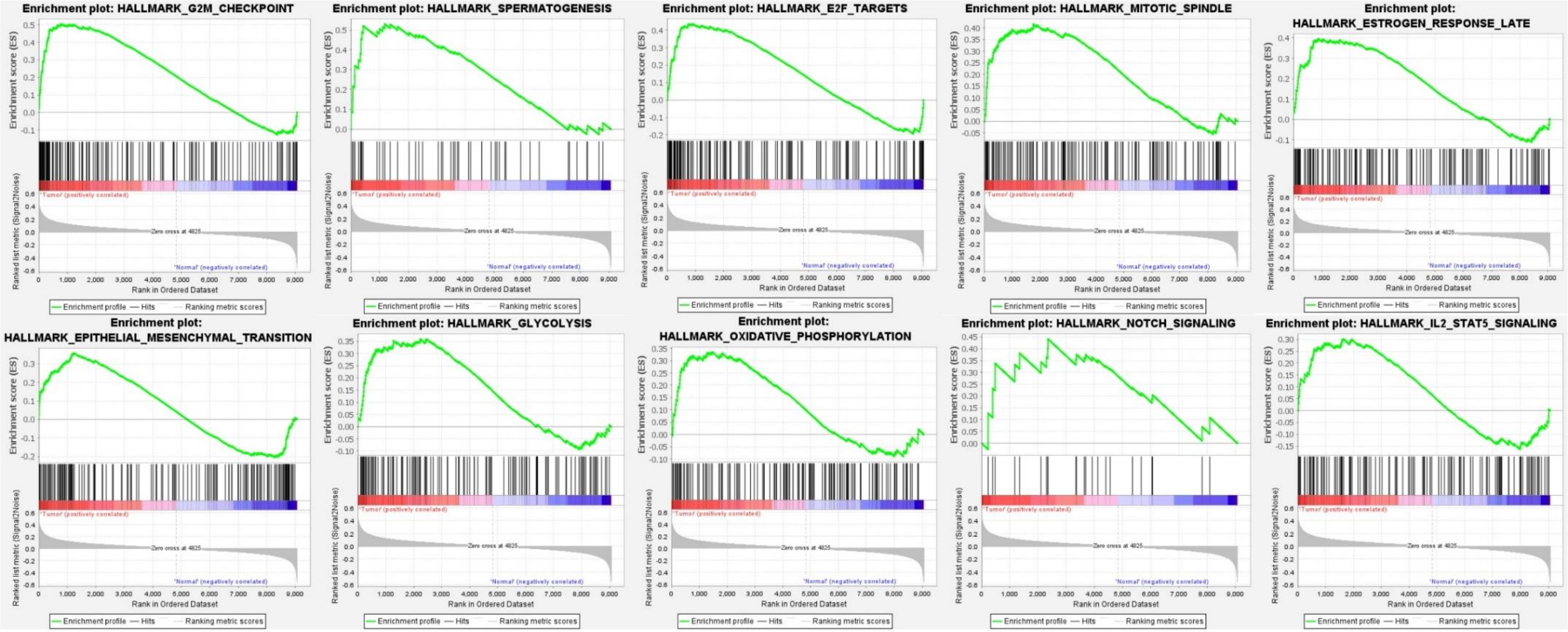

Supplement: Supplementary file 1 [file pharmaceutics-15-02303-s001.zip › pharmaceutics-2574198-supplementary.pdf]
